# Periodontal disease in a patient with Prader-Willi syndrome: a case report

**DOI:** 10.1186/1752-1947-5-329

**Published:** 2011-07-28

**Authors:** Manabu Yanagita, Hiroyuki Hirano, Mariko Kobashi, Takenori Nozaki, Satoru Yamada, Masahiro Kitamura, Shinya Murakami

**Affiliations:** 1 Department of Periodontology, Division of Oral Biology and Disease Control, Osaka University Graduate School of Dentistry, 1-8 Yamadaoka, Suita, Osaka 565-0871, Japan

## Abstract

**Introduction:**

Prader-Willi syndrome is a complex genetic disease caused by lack of expression of paternally inherited genes on chromosome 15q11-q13. The prevalence of Prader-Willi syndrome is estimated to be one in 10,000 to 25,000. However, descriptions of the oral and dental phenotype are rare.

**Case presentation:**

We describe the clinical presentation and periodontal findings in a 20-year-old Japanese man with previously diagnosed Prader-Willi syndrome. Clinical and radiographic findings confirmed the diagnosis of periodontitis. The most striking oral findings were anterior open bite, and crowding and attrition of the lower first molars. Periodontal treatment consisted of tooth-brushing instruction and scaling. Home care involved recommended use of adjunctive chlorhexidine gel for tooth brushing twice a week and chlorhexidine mouthwash twice daily. Gingival swelling improved, but further treatment will be required and our patient's oral hygiene remains poor. The present treatment of tooth-brushing instruction and scaling every three weeks therefore only represents a temporary solution.

**Conclusions:**

Rather than being a direct result of genetic defects, periodontal diseases in Prader-Willi syndrome may largely result from a loss of cuspid guidance leading to traumatic occlusion, which in turn leads to the development of periodontal diseases and dental plaque because of poor oral hygiene. These could be avoided by early interventions to improve occlusion and regular follow-up to monitor oral hygiene. This report emphasizes the importance of long-term follow-up of oral health care by dental practitioners, especially pediatric dentists, to prevent periodontal disease and dental caries in patients with Prader-Willi syndrome, who appear to have problems maintaining their own oral health.

## Introduction

Prader-Willi syndrome (PWS) is a complex multi-system genetic disorder that results from abnormalities in the critical region of chromosome 15q11.2-q13, including paternal interstitial deletion, maternal uniparental disomy and imprinting defects [1,2]. PWS is characterized by infantile hypotonia, poor suck, hyperphagia and subsequent obesity, hypogonadism, mental retardation and various learning disabilities. In addition, PWS is associated with a variety of musculoskeletal abnormalities including scoliosis, short hands and feet, facial dysmorphy, narrow hands with straight ulnar border, delayed bone age and joint hyperlaxity. Characteristics of this syndrome indicate hypothalamic dysfunction, and the incidence of the disorder is estimated at 1 in 10,000 to 25,000 individuals. The disorder can be divided into several stages [1,2]. The neonatal stage is mainly characterized by severe hypotonia and poor suck, which improves with age. Hyperphagia and subsequent obesity present in infancy, and small stature, developmental delay and behavioral problems (rigidity, stubbornness) are manifested during childhood. Adolescence is characterized by incomplete and delayed puberty, infertility, and deterioration of oppositional behavior (persistence of annoying behaviors, tantrums, and obsessive-compulsive behavior). Management of PWS, therefore, requires a multidisciplinary professional, parental and social approach to reduce morbidity and improve quality of life [3].

Previously described oral and dental features of PWS include hypoplastic enamel [4,5], rampant caries [4,5], and dental erosion [6]. With respect to periodontal diseases, only one previous report has documented early-onset periodontitis in a 12-year-old girl with PWS [7]. We report a case of PWS in a 20-year-old man who presented to our facility with severe and localized periodontitis.

### Case presentation

A 20-year-old Japanese man presented with his father to our periodontic clinic at Osaka University Dental Hospital, Japan, because of his poor oral condition. His chief complaint was severely painful gums, which led to crying. He was unable to provide detailed information about when and how this problem had started. His father told us that our patient had thought there was a gingival problem since childhood, had complained of gingival pain for the previous six months, and had been unable to brush his teeth for the last two weeks. Our patient's height was 153 cm and his weight was 62 kg. His medical history included a three-month hospital stay caused by low birth weight and cyanosis just after birth. A clinical diagnosis of PWS had been made on the basis of symptoms such as hypotonia, genital hypoplasia, acromicria (short hands and feet) and genetic testing.

**Figure 1:**
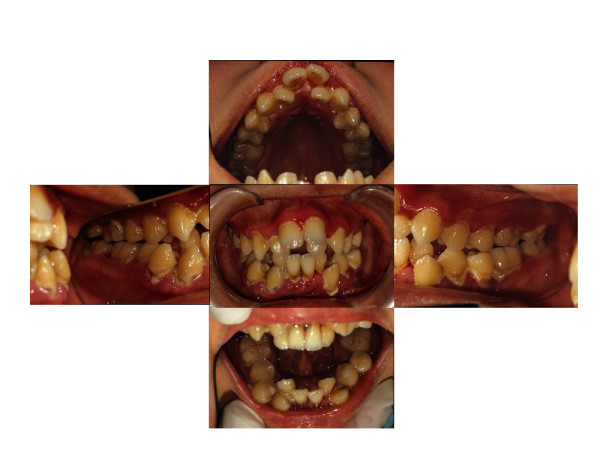
**Intra-oral clinical appearance at initial visit**.

**Figure 2:**
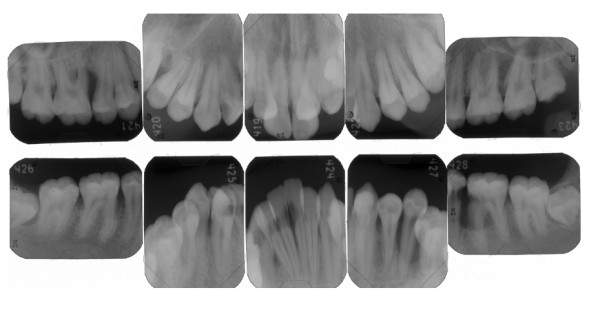
**Baseline full-mouth periapical radiograph**.

An intra-oral examination revealed poor oral hygiene with heavy generalized plaque throughout the permanent dentition. His gingival tissues showed marginal redness, swelling, and food impaction (Figure 1). An apparent anterior open bite, increasing overjet, an anterior crowded arch and malpositioning of the teeth were noted. In addition, circular caries and attrition of the mandibular first molars were present. Pocket depths ranged from 4 mm to 8 mm. Mobility grade 2 was present in the mandibular left second premolar. We tried to measure the pocket depth and clinical attachment level at his first visit. Unfortunately, however, our patient did not allow the pocket measurement because of pain. Thus, we could not perform the conventional pocket measurement and examined only the mesial and buccal/labial pockets. Periapical radiographs disclosed localized vertical bone resorption (mesiolateral of maxillary right first molar and mesiolateral of mandibular left first molar) (Figure 2). Of particular note, the mesiopalatal pocket depth of the maxillary right first molar was 8 mm.

**Figure 3:**
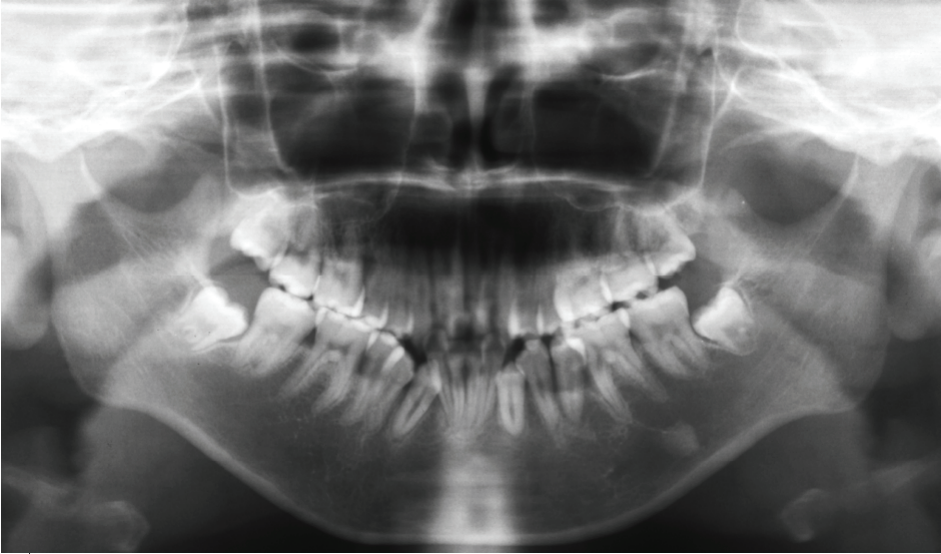
**Panoramic radiograph**.

Based on pocket measurements and an X-ray examination, our patient was diagnosed with localized periodontitis. In addition, our patient exhibited several caries lesions from the mandibular right anterior teeth to the left molars. An orthopantomogram (Figure 3) showed full permanent teeth with unerupted lower third molars. There was no family history of periodontitis.

**Figure 4:**
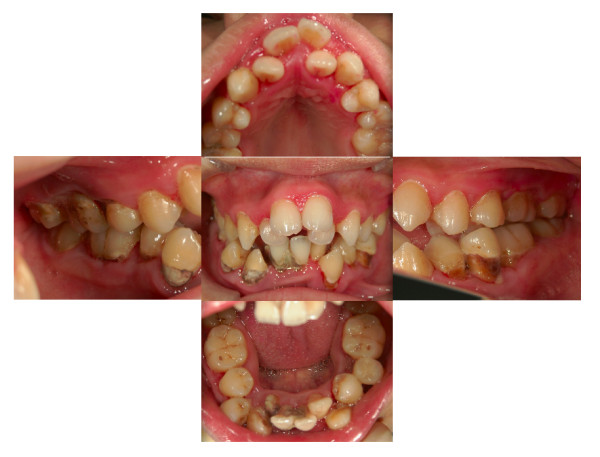
**Clinical image at six months showing some improvement of gingival swelling**.

Advice on periodontal treatment was provided in the presence of one of our patient's parents. This included oral hygiene instructions on how to control plaque using a manual toothbrush (Sam Friend Supersoft #300; Sun Dental, Osaka, Japan), 0.2% w/w chlorhexidine mouth rinse (ConCool F; Weltech, Osaka, Japan) and 1% v/w chlorhexidine gel (ConCool Gelcoat F; Weltech). Professional scaling was also performed to remove supragingival plaque. Our patient kept his treatment appointments with his father every three weeks, but his plaque control was poor. His father told us that our patient was motivated to brush his teeth and did so happily, but sometimes he fell asleep without brushing because of daytime somnolence; a common occurrence in PWS [3]. He refused to allow his parents to help him brush his teeth. Furthermore, malpositioning of the teeth and difficulties with hand and wrist movements inhibited adequate plaque control. After three visits to the clinic our patient had become accustomed to the dental treatment, and subgingival scaling was performed using an ultrasonic scaler. However, active treatments such as root planing and periodontal surgery were not employed because of poor plaque control. Both tooth-brushing instruction (TBI) and subgingival scaling were performed every three weeks. Although some gingival inflammation remained, his gingival swelling and redness were reduced by six months after his first visit (Figure 4).

## Discussion

Most reports on the dental findings in PWS have focused on the presence of rampant caries, tooth wear, delayed tooth eruption, and hypoplastic enamel [4-6]. However, little is known about periodontal diseases in PWS. To the best of our knowledge, the only report in the English literature concerned a case of early-onset periodontitis in a 12-year-old girl with PWS [7]. The present report demonstrates the presence of moderate-to-severe periodontal destruction in a patient with PWS.

Several researchers have reported severe periodontal diseases in patients with systemic genetic disorders such as Marfan syndrome (MFS), Ehlers-Danlos syndrome (EDS) and Down's syndrome (DS), which are accompanied by connective tissue disorders characterized by joint hypermobility and scoliosis. MFS is caused by mutations in the fibrillin-1 gene on chromosome 15q21.1, leading to abnormalities in the connective tissue matrix [8]. Straub *et al*. reported that inflammatory periodontal breakdown in a patient with MFS was caused by abnormalities in the periodontal connective tissue rather than being attributable to the primary biochemical changes of MFS [9]. EDS comprises a heterogeneous group of inherited connective tissue disorders characterized by joint laxity, chronic joint pain, skin hyperextensibility, tissue fragility and scoliosis [10]. EDS can be divided into 11 phenotypes, and periodontal diseases have been reported in EDS types I and VIII. The pathogeneses of these subtypes of EDS are unknown. The periodontal disease in EDS type I is localized, whereas EDS type VIII is characterized by ligneous periodontitis or persistent hyperplastic gingivitis [11]. DS is a chromosomal disorder that results from an extra copy of chromosome 21 (trisomy 21). Patients with DS have a variety of associated medical conditions, including joint hypermobility and ligamentous laxity [12]. The immune alternations in DS, including impaired neutrophil chemotactic function, are responsible for the defensive mechanisms in periodontal disease, which are frequently experienced by subjects with DS [13,14]. As in MFS, EDS and DS, musculoskeletal abnormalities such as scoliosis, joint hyperlaxity, delayed bone age and osteoporosis, are also clinical features of PWS [3]. Although a number of imprinted genes have been mapped to the PWS region at 15q11.2-q13, there is no case normal paternal copy of 15q11-q13 with inheritance consistent with a single mutated gene, suggesting that PWS is a multigenic syndrome [15]. Furthermore, there is no gene related to the connective tissue matrix in the PWS region. The periodontal breakdown demonstrated in this case report may have been the combined result of poor plaque control, crowding, tooth malposition, and occlusal trauma. In particular, the presence of plaque and inflammation in our patient would appear to be a primary etiology, with occlusal imbalances as a contributing factor. Additionally, the interactions of multiple genes affected in PWS may increase the susceptibility to periodontal breakdown via effects on the host immune response; a recent report has shown that an overactivation of the innate immune system and increased chronic low-grade inflammation has been observed in patients with PWS [16]. Periodontal disease in PWS, as shown in this report, is considered to be similar to cases of patients with DS with regard to traumatic occlusion, mouth breathing with an open bite, poor plaque control and immune alternations. Further studies are needed to clarify the relationship between the genetic defect(s) and periodontal disease in PWS.

A recent study that surveyed the orodental characteristics of 15 patients with PWS with early multidisciplinary follow-up at the university hospital reported low incidence of caries and enamel hypoplasia [6]. However, our patient did not receive a multidisciplinary medical follow-up, and it is possible that such follow-up, including dental procedures, could have prevented the development of periodontal disease and caries. Learning disabilities in individuals with PWS mean that prolonged treatments such as periodontal surgery with local anesthesia or oral self-care with several cleaning aids such as tooth brushing, inter-dental brushing and dental flossing, which require tolerance and technique on the part of the patient, are hardly applicable. Thus, oral preventive treatments or interventions from early childhood, such as optimal plaque control and occlusal improvement by orthodontic treatments, should be implemented and may be beneficial for patients with PWS.

## Conclusions

To the best of our knowledge, we describe the first case reported in the literature of periodontal diseases in a patient with PWS with severe malposition of his teeth and traumatic occlusion. Because of the difficulty in oral self-care for patients with PWS, we believe that both medical and dental doctors should be involved in maintaining oral health care of such patients, with early intervention and long-term follow-up.

## Consent

Written informed consent was obtained from the patient for publication of this case report and any accompanying images. A copy of the written consent is available for review by the Editor-in-Chief of this journal.

## Competing interests

The authors declare that they have no competing interests.

## Authors' contributions

MY and SM drafted the manuscript. MY, HH, MK, TN, SY and MK were involved in our patient's care and follow-up. All authors have read and approved the final manuscript.
